# Evaluating Policy, Systems, and Environmental Change Interventions: Lessons Learned From CDC’s Prevention Research Centers

**DOI:** 10.5888/pcd12.150281

**Published:** 2015-10-15

**Authors:** Sally Honeycutt, Jennifer Leeman, William J. McCarthy, Roshan Bastani, Lori Carter-Edwards, Heather Clark, Whitney Garney, Jeanette Gustat, Lisle Hites, Faryle Nothwehr, Michelle Kegler

**Affiliations:** Author Affiliations: Jennifer Leeman, School of Nursing, University of North Carolina at Chapel Hill, Chapel Hill, North Carolina; William J. McCarthy, Roshan Bastani, Fielding School of Public Health and Jonsson Comprehensive Cancer Center, University of California Los Angeles, Los Angeles, California; Lori Carter-Edwards, Gillings School of Global Public Health, University of North Carolina at Chapel Hill, Chapel Hill, North Carolina; Heather Clark, Whitney Garney, Texas A&M School of Public Health, College Station, Texas; Jeanette Gustat, Tulane University School of Public Health and Tropical Medicine, Prevention Research Center, New Orleans, Louisiana; Lisle Hites, University of Alabama at Birmingham Prevention Research Center, University of Alabama at Birmingham, Birmingham, Alabama; Faryle Nothwehr, University of Iowa Prevention Research Center for Rural Health, College of Public Health, University of Iowa, Iowa City, Iowa; Michelle Kegler, Emory Prevention Research Center, Rollins School of Public Health, Emory University, Atlanta, Georgia.

## Abstract

**Introduction:**

The field of public health is increasingly implementing initiatives intended to make policies, systems, and environments (PSEs) more supportive of healthy behaviors, even though the evidence for many of these strategies is only emerging. Our objective was 3-fold: 1) to describe evaluations of PSE-change programs in which the evaluators followed the steps of the Centers for Disease Control and Prevention’s (CDC’s) *Framework for Program Evaluation in Public Health,* 2) to share the resulting lessons learned, and 3) to assist future evaluators of PSE-change programs with their evaluation design decisions.

**Methods:**

Seven Prevention Research Centers (PRCs) applied CDC’s framework to evaluate their own PSE-change initiatives. The PRCs followed each step of the framework: 1) engage stakeholders, 2) describe program, 3) focus evaluation design, 4) gather credible evidence, 5) justify conclusions, and 6) ensure use and share lessons learned.

**Results:**

Evaluation stakeholders represented a range of sectors, including public health departments, partner organizations, and community members. Public health departments were the primary stakeholders for 4 of the 7 evaluations. Four PRCs used logic models to describe the initiatives being evaluated. Their evaluations typically included both process and outcome questions and used mixed methods. Evaluation findings most commonly focused on contextual factors influencing change (process) and the adoption or implementation of PSE-change strategies (outcome). Evaluators shared lessons learned through various channels to reach local stakeholders and broader public health audiences.

**Conclusion:**

*Framework for Program Evaluation in Public Health* is applicable to evaluations of PSE-change initiatives. Using this framework to guide such evaluations builds practice-based evidence for strategies that are increasingly being used to promote healthful behaviors.

## Introduction

Physical inactivity, tobacco use, and other unhealthy behaviors increase risk for numerous chronic conditions and are among the leading contributors to morbidity and mortality (1–3). Efforts to change these behaviors will have limited success as long as policies, systems, and environments (PSE) are unsupportive of healthy behaviors (4,5). To maximize impact on population health, the Centers for Disease Control and Prevention (CDC) increased its investment in PSE-change interventions (6,7). However, despite the almost universal acceptance that changes in PSEs will improve healthful behaviors, the hard evidence for their effectiveness is just beginning to emerge (8–11). The good news is that with CDC’s investment in PSE change comes an opportunity to build the evidence base for PSE interventions. To take advantage of this opportunity, PSE-change initiatives should include rigorous process and outcome evaluations (12).

PSE interventions are challenging to evaluate because they are often complex (13). PSE interventions require the involvement of many diverse stakeholders, each of which brings different resources, needs, and values to the project. Therefore, the design of the intervention and its evaluation usually cannot be predetermined; instead they evolve over time to fit stakeholder priorities (14,15). Another evaluation challenge is that PSE interventions are often designed to achieve their effects through interactions with multiple causal factors over extended periods, thus making results difficult to interpret (13,16).

The CDC Prevention Research Centers (PRC) program funds a network of universities to conduct prevention research and partner with public health practitioners and local communities to design, implement, and evaluate interventions to prevent disease (17). The objective of our study was to describe how 7 PRCs used CDC’s *Framework for Program Evaluation in Public Health* (18) to evaluate PSE interventions and to share the resulting lessons learned, with the goal of assisting future evaluators considering how to assess PSE-change initiatives.

## Methods

We invited 37 PRCs funded from 2009 through 2014 to contribute to this study if they had evaluated a PSE initiative. In 2014, seven PRCs provided information about their PSE evaluations in a series of conference calls organized by the 6 steps of the CDC framework. 

The Emory University PRC received a contract from the Mississippi State Department of Health to evaluate a community-based initiative focused on reducing stroke and cardiovascular disease in the Mississippi Delta (19). The initiative funded mayors’ offices, federally qualified health centers, and nonprofit organizations to implement community-driven PSE changes that promoted physical activity, nutrition, tobacco use prevention and cessation, and chronic disease self-management.

The Texas A&M University PRC evaluated local health advisory commissions that were established in 4 rural counties, with members appointed by county government. The PRC also evaluated the interventions that the health advisory commissions implemented in their counties to increase access to physical activity (20).

The Tulane University PRC evaluated its Partnership for an Active Community Environment (PACE) project (21). Tulane partnered with neighborhood community groups and the City of New Orleans to implement the PACE project, part of which consisted of creating a 6-block walking path connecting a community park to a business corridor. The Tulane PRC paid for 2 blocks of the path and partnered with the city to complete the other 4 blocks.

The University of Alabama at Birmingham (UAB) PRC supported their local health department in designing, implementing, and evaluating a policy initiative that encouraged convenience and corner store owners in low-income, predominantly African American communities to voluntarily display point-of-sale tobacco warnings.

The University of California, Los Angeles (UCLA) PRC evaluated its WORKING Program: Working Out Regularly Keeps Individuals Nurtured and Going (22). WORKING promoted healthy nutrition and physical activity at worksites in Southern California. Worksites were predominantly health and human services agencies that employed high proportions of racial/ethnic minority people. Each worksite selected intervention strategies best suited to its organization from a menu of options (eg, stair prompts, vending machine policies).

The University of Iowa (UI) PRC for Rural Health received a contract from the Iowa Department of Public Health to assist in evaluating its CDC-funded Community Transformation Grant (CTG) (23). The Iowa Department of Public Health provided sub-awards to 26 communities (15 rural, 11 urban) to carry out community assessments; communities used findings to inform interventions related to nutrition, physical activity, tobacco, and other health concerns.

The University of North Carolina at Chapel Hill (UNC) PRC received a contract from the North Carolina Division of Public Health to evaluate the impact of the CTG projects on health equity (24). The CTG projects focused on improving healthy eating, physical activity, and tobacco-free living across North Carolina.

The first step of the CDC framework is to engage stakeholders; stakeholder input helps generate credible, useful findings and can increase the evaluation’s cultural appropriateness (18). The second step is to describe the program; this description should then inform subsequent evaluation decisions (18). The third step is to focus the evaluation design; this is an iterative planning process for stakeholders to determine the evaluation purpose and approach (18). The fourth step in the CDC framework is to gather credible evidence, information that evaluation stakeholders perceive as “believable and relevant for answering their questions” (18). The fifth step is to justify conclusions; this step includes appropriately analyzing data, summarizing and interpreting findings, and making judgments or recommendations based on the data (18). The final step of the CDC framework is to ensure use and share lessons learned. This step primarily focuses on providing useful information to evaluation stakeholders; it also includes activities to share lessons learned more broadly (18).

## Results

### Engage stakeholders

Each PRC project and community was unique; however, we found similarities in the types of stakeholders involved (Table 1 and Table 2). Three PRCs were external evaluators contracted by a state department of public health, which was the primary intended user of the evaluation. Four PRCs were internal evaluators; the primary intended users of the evaluations were the organization responsible for implementing the intervention (eg, worksite, local public health department), the project steering committee, and community partners. In a few cases, the primary intended users of the evaluation were also members of the project’s community advisory board.

**Table 1:** Description of Prevention Research Center Evaluations of Initiatives to Change Policies, Systems, and Environments

Description	No.	Emory	Texas A&M	Tulane	UAB	UCLA	UI	UNC
**Evaluation client**								
State department of public health	3	X					X	X
Not applicable (internal evaluator)	4		X	X	X	X		
**How program was described**								
Logic model	4	X	X				X	X
Conceptual framework	3	X		X		X		
Logic mapping	1				X			
**Study design**								
Cross-sectional	4	X	X	X			X	
Pre–post assessment	3		X	X		X		
Longitudinal follow-up	2		X			X		
Case study	1							X
Control group	1					X		
Comparison group	1			X				
**Data sources**								
Surveys	5	X	X	X		X		X
Observation	4		X	X	X	X		
Interviews	3	X	X					X
Document review	3	X	X				X	
**Analysis methods**								
Mixed methods	5	X	X	X	X	X		
Qualitative	1							X
Quantitative	1						X	
Stakeholders involved in justifying conclusions	5	X	X	X			X	X
**Focus of findings**								
Adoption/ implementation of PSE strategies	5	X			X	X	X	X
Contextual factors influencing PSE change	5	X	X		X	X		X
Individual behavior change	2			X		X		
Methodological issues	1						X	
**Sharing lessons learned**								
Evaluation report	7	X	X	X	X	X	X	X
Meeting/presentation to stakeholders	4	X	X	X			X	
Manuscript for peer-reviewed publication	7	X	X	X	X	X	X	X
Conference presentation	6		X	X	X	X	X	X

Abbreviations: PSE, policy, systems, and environmental; UAB, University of Alabama at Birmingham; UCLA, University of California, Los Angeles; UI, University of Iowa; UNC, University of North Carolina at Chapel Hill.

**Table 2:** Primary Intended Users of Evaluation, Key Stakeholders, Evaluation Questions, and Indicators for PRC Evaluations of Policy, Systems, and Environmental Change Initiatives

PRC/PSE-Change Initiative	Primary Intended User and Other Key Stakeholders	Evaluation Questions	Indicators
**Emory University**			
Mississippi State Department of Health conducted a community-based initiative to reduce stroke and cardiovascular disease in the Mississippi Delta.	• Mississippi State Department of Health (primary user) • Program staff • Grantee organizations	• What steps were taken toward PSE change as a result of the initiative? • How many and what types of PSE changes were made as a result of the initiative? • What facilitated and inhibited progress in creating PSE change in various sectors?	• Steps in PSE-change process • Number and type of PSE changes made • Capacity for PSE work • Barriers/ facilitators to implementation
**Texas A&M University**			
Local health advisory commissions in 4 rural counties implemented county-specific interventions to increase access to physical activity.	• Community partners (primary user) • County health resource centers staff • County and city governments • Community members	• Does community health development improve community capacity? • How do local communities operationalize dimensions of community capacity to implement a successful intervention? • What are the benefits and barriers to participation in a health commission? • How do commission initiatives, such as supporting a health resource center, increase access to health care and social services in rural areas?	• Partnership measures • Community capacity dimensions • Physical activity intervention data • Community health development process measures
**Tulane University**			
Partnership for an Active Community Environment (PACE) project included creating a 6-block walking path connecting a community park to a business corridor.	• Steering committee (primary user) • Residents	Did the environmental intervention make a difference in people’s physical activity levels?	• Self-reported neighborhood residents’ physical activity level • Number of people observed engaging in physical activity
**University of Alabama at Birmingham**			
Policy initiative encouraged convenience and corner store owners in low-income, predominantly African American communities to voluntarily display point-of-sale tobacco warnings.	• Local health department (primary user) • Convenience and corner store owners • Partner organizations	Can voluntary policy implementation be an effective tool for policy intervention?	Acceptance and placement of point-of-sale tobacco warnings
**University of California, Los Angeles**			
Working Out Regularly Keeps Individuals Nurtured and Going (WORKING) Program promoted healthy nutrition and physical activity at worksites in Southern California.	Worksite leadership and program champions	• How many and what type of nutrition and physical activity policies and practices were adopted and implemented by the organization? • Were changes sustained?	• Number and type of organizational health promotion policies and procedures adopted and implemented • Barriers and facilitators to implementation
**University of Iowa**			
Iowa Department of Public Health funded 26 communities to carry out community assessments as part of their CTG project. Findings were used to inform interventions related to nutrition, physical activity, tobacco, and other health concerns.	Iowa Department of Public Health	• In the CTG-identified strategic directions, which PSEs are most often identified on the CDC-developed CHANGE tool as in need of improvement in the community at large and in the targeted worksites? • Which PSEs are considered as not applicable to the setting? • What differences and similarities are found between the CHANGE tool assessment in rural and urban counties?	Scores for physical activity policy and environmental factors in the community at-large and in targeted worksites
**University of North Carolina at Chapel Hill**			
North Carolina Division of Public Health’s CTG project focused on improving healthy eating, physical activity, and tobacco-free living.	• North Carolina Division of Public Health (primary user) • CTG project staff and coalition members • Collaborating agencies • Community members	• Which CTG project strategies work in addressing health disparities? • For whom do the CTG project strategies work? • Under what conditions do the CTG project strategies work to reduce health disparities? • What is the impact of CTG project interventions on reducing health disparities among low-income and rural groups? • How do people from health-disparate populations experience systems and environmental changes related to CTG project improvements?	Perceptions of: • Barriers/facilitators • Engagement of stakeholders • Distribution of power • Implementation responsiveness to community beliefs, norms, practices • Potential to promote health equity • Distribution equity of reach, adoption, implementation, and effectiveness

Abbreviations: CDC, Centers for Disease Control and Prevention; CHANGE, Community Health Assessment aNd Group Evaluation; CTG, Community Transformation Grant; PRC, Prevention Research Center; PSE, policies, systems, and environments.

PRCs reported various stakeholder roles in evaluation. Evaluation stakeholders served both as advisors and as collaborative partners. Stakeholder involvement during the early phases of evaluation included providing input about appropriate evaluation participants and effective ways to access them, evaluation questions, data collection tools, and data collection plans. Nearly every PRC reported stakeholder involvement during the design phase. Stakeholder evaluation roles in mid-project focused primarily on data collection; 5 PRCs involved stakeholders directly in data collection. In later stages, PRCs most commonly engaged stakeholders in interpreting results and disseminating evaluation findings. Six participating PRCs reported such stakeholder involvement during the evaluation’s final stages. To communicate with their stakeholders, most PRCs reported holding regular meetings or conference calls.

### Describe the program

Approaches to developing models for describing a program differed according to whether the PRC was an internal evaluator or external evaluator (Table 1). The 4 internal evaluator PRCs were involved in developing and describing the program from its inception: one developed a logic model, two developed a conceptual framework to guide the intervention, and one used an iterative process to turn work plans into a flow-chart style logic map.

The 3 external evaluator PRCs needed to describe a program with which they were unfamiliar. All three reviewed program documents and met with stakeholders to develop an understanding of the program. Two used this information to develop a logic model. The third used a logic model created by the program staff before contracting with the PRC to do an evaluation; the PRC also developed a conceptual framework to guide the evaluation.

### Focus the evaluation design

PRCs and their evaluation stakeholders selected a range of process and outcome evaluation questions; process evaluation questions focused on topics such as context, PSE strategy selection, PSE-change process, and program reach. Outcome evaluation questions covered topics such as adoption and maintenance of PSE changes, community capacity for change, residents’ access to health care, individual behavior change, and impact of changes on health disparities (Table 2).

Five PRCs used evaluation designs that included mixed methods of data collection (Table 1). Four used a cross-sectional design; for example, Emory conducted stakeholder interviews and surveys of grantees’ community partners. Three PRCs used pretest and posttest designs to assess changes in study outcomes; for example, Tulane used surveys and observations of neighborhood residents to assess physical activity levels before and after the installation of a walking path. Two PRCs that used pretest and posttest designs also included longitudinal follow-up after the intervention ended. One PRC used a case study design. One PRC had a comparison group, and another had a control group for its study. For example, UCLA conducted a cluster-randomized, wait-list–controlled evaluation of its program. Given resource constraints, most PRCs that assessed intervention effects over time used uncontrolled pre- and post-cross-sectional surveys.

### Gather credible evidence

PRCs and their evaluation stakeholders selected a range of indicators for their evaluation questions (Table 2). Process indicators included barriers and facilitators and completion of steps in the PSE-change process. Outcome indicators included the number and type of policies adopted and implemented and self-reported physical activity levels. Five PRCs used mixed methods, and five used multiple data collection methods. Five PRCs used surveys, four conducted direct observations, three conducted interviews, and three reviewed project documents (Table 1).

### Justify conclusions

The study PRCs used several data analysis methods and a variety of approaches to summarize and interpret findings: 5 PRCs used mixed data analysis methods, one PRC used quantitative methods exclusively, and one PRC used qualitative methods exclusively (Table 1). Three PRC evaluations were primarily descriptive. One of these PRCs used the CDC’s Community Health Assessment aNd Group Evaluation (CHANGE) Action Guide (25) to determine the PSE-change strategies that were most and least prevalent in 26 intervention communities. Two PRCs assessed the proportion of targeted organizations (eg, stores, churches) that adopted particular PSE changes (eg, point-of-sale tobacco signage, church garden). Two PRCs looked for significant differences between intervention and comparison or control groups; one of these PRCs also categorized intervention sites as high- or low-performance. Five PRCs engaged stakeholders in interpreting results or generating recommendations, or both (Table 1).

All PRCs generated findings and recommendations that could be used for program improvement or to inform future initiatives (Table 1). Five PRCs reported findings or made recommendations about the adoption or implementation of PSE changes (eg, community-based organizations were able to make organizational-level PSE changes within 6 to 12 months) and the contextual factors that influenced successful PSE changes (eg, an organization’s history of supporting wellness initiatives). Two PRCs reported findings focused on behavior change resulting from PSE initiatives (eg, residents’ activity levels increased after a walking path was installed in the neighborhood). Finally, one PRC also generated methodological recommendations (eg, the need to adapt community-level assessment tools to be applicable to rural communities).

### Ensure use and share lessons learned

The PRCs employed 4 kinds of strategies to ensure use of the evaluations and share lessons (Table 1). First, all 7 PRCs produced evaluation reports, which were reports required by funders or reports tailored to provide useful and timely information for stakeholders on implementation and outcomes. Three reports were coauthored with community partners, which helped to ensure accuracy and strengthen partnerships. Second, 4 PRCs held meetings with stakeholders to review findings and discuss recommendations. Third, all 7 PRC’s produced manuscripts for peer-reviewed journals to disseminate findings and lessons learned. Six have been published or are forthcoming (19–24), and one manuscript was under review at the time of this writing. Finally, 6 PRCs presented evaluation findings at regional or national conferences attended by researchers and practitioners.

## Discussion

This article describes how 7 PRCs evaluated PSE-change initiatives, focusing on how PRCs carried out each step of the CDC framework. Understanding how best to evaluate PSE initiatives is important, given the significant investment of public health resources in such initiatives (4–7,26), despite the modest evidence base for the effectiveness of many PSE intervention strategies (8,12). 

Evaluators need to engage a broad diversity of stakeholders to address the complex, multisectoral nature of PSE change. Evaluation stakeholders were from a range of sectors, including public health departments, community organizations, and community members. PRCs most commonly identified the organization responsible for implementing the initiative as the primary intended user (eg, state health department, worksite). Most PRCs actively engaged with intended users throughout the intervention process, which is one of the most effective ways to ensure that findings will be used.

It is appropriate for PSE evaluation outcomes to focus on changes in policies and environments, provided that an established link exists between those structural changes and health outcomes. For example, increasing the unit price of tobacco is known to reduce tobacco use (11). Therefore, the evaluation needs to document only that the unit price has increased and does not need to replicate research on its effectiveness. However, in many areas additional studies are needed to understand better the relationship between implementation of PSE changes and desired health outcomes (8–10). The most common outcome of interest in these evaluations was the adoption, implementation, or maintenance of PSE changes. Few evaluations assessed behavior changes resulting from PSE changes, and none assessed health outcomes. This finding is consistent with the findings of other published evaluations of PSE-change initiatives. For example, Soler et al reported outcome evaluation findings from 6 PSE-change initiatives funded by CDC’s Communities Putting Prevention to Work program (27); most of these initiatives focused on changes to policies and environments (28–31), and none assessed changes in health behaviors or health outcomes. It is challenging to assess long-term changes resulting from PSE interventions when funding is typically limited to a few years. As the initial funding ends, public health departments and community organizations often prioritize sustainability and strive to maintain program components over evaluation activities. Future research should focus on identifying innovative methods and evaluation designs for linking PSE changes to existing data on outcomes of interest. Another option is to pursue complementary funding streams and collaborations to support evaluations. For example, a public health department could partner with a research university to seek foundation funding to evaluate a PSE initiative.

Furthermore, it is often difficult to differentiate between policy, systems, and environmental approaches; for example, although CDC has referred to PSE change in many of its programs, it has also described policy change as a type of environmental approach (32). Lieberman and colleagues (33) use the term *structural approach* to encompass multiple types of strategies. Using clearer and more consistent definitions of what is meant by PSE or structural change initiatives when designing and describing programs may help evaluators design studies that can tease out the impact of different components or types of interventions and strengthen our knowledge base about such strategies.

Three PRCs also assessed the interventions’ impact on capacity and partnerships. Because PSE change is a new focus for many public health practitioners, capacity and partnership building may be an important precursor to intervention planning and implementation. Six PRCs also gathered evidence on contextual factors that may impede or facilitate implementation, evidence that is critical to strengthening PSE interventions.

PRCs were often limited in the type of research designs used. Most were cross-sectional, and only one evaluation included a randomized control group with longitudinal follow-up. Such modest designs are understandable given funding constraints and the resource-intensive nature of rigorous evaluation of large-scale, community-based PSE-change initiatives (13). However, to build the evidence base for PSE-change strategies, there is a need for stronger collaborations that involve evaluators early enough to measure baseline rates and to use comparison or control groups. CDC and other major public health funders should prioritize funding of more rigorous evaluations of PSE-change initiatives.

This article provides an overview of multiple evaluations; this format necessarily gives limited detail about any one study and simplifies methodological issues raised by each. Additionally, we found differences in PSE-change initiatives, their level of funding for evaluation, and the PRC’s role as internal or external evaluator. We provided a brief description of each initiative and noted each PRC’s role, but our small sample did not allow us to explore the breadth of these issues or look for relationships between these characteristics and resulting evaluation decisions.

Despite these limitations, the experience of these 7 PRCs offers useful lessons for evaluations of PSE-change initiatives. To our knowledge, this is the first article to describe how all 6 steps of the CDC framework can be applied to PSE interventions. Less frequently described steps in this framework may be particularly relevant to PSE initiatives. For example, attention to describing the program may be helpful in evaluations of complex interventions where change is expected to occur incrementally and where attribution to any one intervention is difficult (13). The final step of ensuring use and sharing lessons learned is critically needed in this area (8–10). Six PRCs published their evaluation findings, contributing to the evidence base on how to translate PSE strategies into changes in practice (19–24). Using the CDC framework to guide evaluations of PSE-change initiatives helps evaluators build practice-based evidence for the growing number of PSE-change strategies being used to promote health.
